# Economic Costs Associated with Motorbike Accidents in Kathmandu, Nepal

**DOI:** 10.3389/fpubh.2016.00273

**Published:** 2016-12-15

**Authors:** Diksha Sapkota, Bihungum Bista, Shiva Raj Adhikari

**Affiliations:** ^1^Kathmandu University School of Medical Sciences, Dhulikhel, Nepal; ^2^Nepal Health Research Council, Ministry of Health and Population, Kathmandu, Nepal; ^3^Patan Multiple Campus, Tribhuwan University, Kathmandu, Nepal

**Keywords:** accidents, DALY, direct cost, indirect cost, motorbike

## Abstract

**Background:**

Road traffic accidents, considered as global tragedies, are in increasing trend; however, the safety situation is very severe in developing countries incurring substantial amount of human, economic, and social costs. Motorcycle crashes, the commonest form, occur mostly in economically active population. However, there is limited number of studies on economic burden of motorcycle crashes. This study aims to estimate the total cost and disability-adjusted life years (DALYs) lost due to motorbike accidents among victims of Kathmandu Valley.

**Materials and methods:**

Retrospective cross-sectional study was conducted among the patients with a history of motorbike accidents within the past 3 to 12 months from the date of data collection. Interview was conducted using *pro forma* among 100 victims of accidents and their caregiver in case of death from November 15, 2014 to May 15, 2015. Cost estimation of motorbike accident was done based on human capital approach. Data collection tool was pretested, and collected data were analyzed using SPSS and Microsoft Excel.

**Results:**

Males (79%) belonging to the economically productive age group shared the highest proportion among total accidents victims. Most common reason for accidents was reported to be poor road condition (41%). Indirect cost was found to be significantly higher than direct costs, highlighting its negative impact on economy of family and nation due to productivity loss. Total DALYs lost per person was found to be 490 years, and national estimation showed large burden of motorbike accidents due to huge DALY loss.

**Conclusion:**

For low-resource countries such as Nepal, high economic costs of motorbike accidents can pose additional burden to the fragile health system. These accidents can be prevented, and their consequences can be alleviated. There is an urgent need for reinforcement of appropriate interventions and legislation to decrease its magnitude and its associated grave economic consequences so as to nib this emerging epidemic in the bud.

## Introduction

According to World Health Organization, about 1.24 million people die every year as a result of road traffic injuries (RTC), and it is a leading cause of death among young people aged 15–29 years (1). In highly motorized countries, RTC mostly involve car drivers, while in certain countries of Asia, majority of them include motorcycle riders (2).

In Nepal, motorbike constitutes the highest share (77.9%) of motorized vehicles (3). Motorcycles are considered to be the most dangerous form of motorized transportations, and the risk of its accidents is further heightened by poor road condition coupled with crowded roads in Kathmandu Valley. Considering its easy maneuverability and speed, victims of motorcycle crash undergo more movement and impact; thus, they sustain more severe injuries (4).

The annual costs of road traffic crashes in low- and middle-income countries are estimated to be between US$65 and $100 billion, which is more than the total annual amount received for development aid (5). The estimated annual national loss from road accidents is more than £9 million (6), and a hospital-based study from Nepal showed that a single injury case cost US$ 126.2 (7). It is estimated that road traffic crashes annually cost between 1 and 1.5% of gross national product in low-income and middle-income countries, putting significant strain on health-care budgets (8). In 2011/2012, more than 12,000 injuries and 1,863 casualties have been reported in Nepal. Of them, 35% belonged to the economically active population of 16–35 years age group, which is bound to adversely affect the economy of the family and nation (6, 9). It is worth to be noted that only those accidents involving deaths, severe injury, or unsolved disputes are reported to the police, while a fairly large number of accidents are settled at the accident site with mutual understanding of both parties, leading to under-representation of accident cases since only reported cases are reflected in national statistics (8, 10).

Its epidemic is largely influenced by choices at individual and policy levels, which suggests that this problem can be controlled, and this further prompts the need of study of road traffic accidents (RTA) from public health and economics perspective. One effective way of bringing the innovative interventions to increase road safety and hence decrease road accidents is to draw attention of governments and policy makers by providing concrete evidence on costs incurred. All the levels of society ranging from individual to societal level are affected by motor vehicle crashes in many ways. For example, all medical care costs are borne by individuals and their families, and any diversion of resources in treatment and rehabilitation are borne by the society. Significant cost is associated with lost productivity by victim and caregivers, which creates economic hardship for whole family due to loss of income coupled with other additional expenditure. A nation not only loses a productive member of the society but also more resources are used in treatment and rehabilitation of such victims. Many families are driven into poverty due to additional cost of prolonged medical care, the loss of a family breadwinner, or the requirement of extra funds to care for people with disabilities. Road crash survivors, their families, friends, and other caregivers often suffer adverse social, physical, and psychological effects that significantly reduce their quality of lives.

Studies aimed at cost estimation are still scarce in developing countries, and the ones that are done are basically crude estimates based on secondary data available from insurance agencies and from hospital bed occupancy. Primary reason for this gap is the lack of proper registry of accident victims and other necessary data needed for conducting large representative survey. There is troublesome gap in information regarding costs associated with long-term consequences of accidents such as disability, rehabilitation, long-term care, and year of life lost (YLL) due to premature deaths. These studies have further limitations such as lack of cost components and unclear methodology for cost estimation.

Hence, in order to overcome these limitations, a strong need was felt to make comprehensive cost estimation by contacting and including people with different types of injuries. Furthermore, very few studies cover the productivity loss of injured person and/or of care takers resulting in a wide discrepancy between the actual cost incurred and the estimated cost. Attempt was made to show accurate representation of the burden of RTA by calculating disability-adjusted life years (DALYs) lost, which can be taken as a reference for conducting further studies and making comparison. This study will help to guide policy makers for designing cost-effective safety measures and management by showing the most accurate economic burden associated with motorbike accidents. This study aimed to take advantage of comprehensive analysis of cost based on human capital approach that is considered to be the most effective way of cost estimation for developing countries and thereby tackle the limitations of existing literatures having a narrow coverage of the costs incurred (11, 12).

## Materials and Methods

### Study Design

This is retrospective cross-sectional study.

### Study Setting

Literatures were reviewed and data were analyzed, which showed that there is continuous increase in number of vehicles registered in Bagmati zone (3). This increasing number of vehicles put additional strain to the already crowded roads of Bagmati zone, especially in Kathmandu Valley. In the last five fiscal years, it was seen that almost 80% of the registered vehicles were motorbikes.

Analyzing the Nepal traffic records, it was evident that out of the total number of vehicles involved in accidents, 33–40% of accidents were of motorbikes. Similarly, out of the total motorcycle accidents of Nepal, more than 50% (56–62%) occurred only in Kathmandu Valley. This justified the selection of Kathmandu Valley for cost estimation of motorbike accidents, and it can represent the true picture of total costs (TCs) sustained in motorbike accidents (3).

### Study Duration

The study duration was 6 months (from November 15, 2014 to May 15, 2015).

### Study Population

The study population comprised victims of RTA, who were enrolled in the study based on the following inclusion criteria: (i) had met with accidents at least 3 months prior to the initiation of the study and within 12 months from the date of interview, (ii) discharged at least a month prior to the initiation of study, and (iii) currently staying in Kathmandu Valley.

### Sample Size

Sample size was purposively selected, i.e., 100 victims of motorbike accidents based on the financial and time limitations. This sample size has been estimated considering it as adequate for application of inferential statistics. A total of 110 respondents were approached, and 10 were excluded from the analysis as they could not complete the interview, thus giving the response rate of 91%.

### Sampling Technique

A list of the people meeting the eligibility criteria was prepared from insurance claims, hospital records, personal acquaintances, etc. The people were then selected randomly from the list and contacted *via* phone to schedule an interview, and the interview was conducted on planned date.

### Data Collection Tools and Techniques

Both primary and secondary data were collected as follows:

- Secondary data: documents, traffic police records, hospital records, bills, national reports, and reviews were reviewed to get the snapshot of the key findings and exiting situation. Medical records and hospital bills were checked to get the cost that is associated with direct medical cost (DMC) wherever feasible.
- Primary data: victim and caretakers were interviewed. In case of death of the victim, the kin involved in care or close acquaintances were interviewed.

Semi-structured questionnaire was developed to assess costs associated with RTC and several sociodemographic factors.

### Data Management and Analysis

Collected data were thoroughly checked for completeness and accuracy. Survey data were entered, compiled, edited, and analyzed using MS Excel and Statistical Package for Social Sciences version 16. Descriptive statistics such as mean, percentage, and SD were used to estimate the cost according to the type of injuries. Costs calculation, graphical presentations, and DALYs estimation were done in MS Excel.

### Cost Calculation

Gross output methodology that employs human capital approach was used to estimate economic costs, considering the fact that it has been preferred for developing countries by Transport Research Laboratory of the United Kingdom and Asian Development Bank (ADB) under the ADB-Association of Southeast Asian Nations regional road safety project (11, 12). All types of costs were presented in average forms in the units of Nepalese rupees and US dollars. This method considers only work performed and ignores the value of leisure. This does not include the subjective costs such as grief, pain, and suffering and provides the minimum value for RTA in a country.

Road accidents were classified into the following three categories (11, 12):

Fatal injury: on spot death or death of victim within 30 days of accident due to the causes directly related to the accident or injuries.
Serious injury: no death but the victim is seriously injured and requires at least an overnight stay in a hospital.
Slight injury: no death or serious injury but victim is slightly injured, requiring medical treatment without any overnight stay in a hospital.

#### Direct Cost Calculation

Direct medical costs included the costs incurred in hospitals such as bed charge, operation charge, medicines, laboratory investigation, rehabilitation cost, and cost of follow-up care. Non-direct medical costs included costs incurred on food, transportation, police investigation, insurance, vehicle repair, vehicle purchase, and legal cost. Cost for both victims and caretakers were taken into account, and total direct cost (TDC) was calculated.

$$\begin{array}{ll} \text{Average direct cost} & =\text{Average direct medical cost}\\ & \;+\text{Average non-direct medical cost} \end{array}$$

$$\begin{array}{ll} \Sigma \left(\text{TDC}/\text{​}N\right) & =\Sigma \left(\text{DMC}/\text{​}N\right)\\ & \;+\Sigma \left(\text{NDMC}/\text{​}N\right)(\text{where}\;N\;\text{is no.}\;\text{of cases}) \end{array}$$

#### Indirect Cost (IC) Calculation

Costing lost output: casualty survey and data from economic survey by Government of Nepal were used for per capita income. For individual older than 15 years, considering them as economically active group, average daily wage rate, i.e., Rs. 318 was used for calculating productivity loss, if the individual is unemployed or his/her monthly income is not known (13).

$$\begin{array}{l} \text{Lost output}(\text{serious})=[\{(\text{no}.\;\text{of in-patient days and days}\\ \;\text{visiting medical facilities}+\text{no}.\text{ of days at home recovering}\\ \;\text{from injuries})\;\times \;(\text{average wages of casualty})\}\\ \;+\;\{(\text{no}.\text{ of days spent caring for casualty by caretaker})\\ \;\times (\text{average wages of caretaker})\}] \end{array}$$

$$\begin{array}{l} \text{Lost output}(\text{slight})\;=\;[\{(\text{no}.\text{ of days visiting medical facilities}\\ \;+\text{no}.\;\text{of days at home recovering from injuries})\\ \;\times (\text{average wages of casualty})\}\;+\;\{(\text{no}.\text{ of days spent caring}\\ \;\text{for casualty by caretaker})\;\times \;(\text{average wages of caretaker})\}] \end{array}$$

Indirect cost (productivity loss) was calculated for both victims and caretakers in case of survivors according to their days of leave from work.

In case of fatalities, productivity loss was calculated using the formula (9):

$$\text{Loss}=\Sigma \frac{W{\left(1+g\right)}^{i}}{{\left(1+r\right)}^{i}}$$

where *W* is the annual per capita gross domestic product (Rs. 699.19), *g* is the annual economic growth rate (4.1%, i.e., 0.041) (13), *r* is the discount rate (standard value given by Global Burden of Disease Study is 0.03) (14), and *i* is the average number of years lost due to fatal accidents (age of people died subtracted from standard life expectancy, i.e., 71 years).

### Calculation of DALYs

Disability-adjusted life years are the sum of years of life lost and years of life lived with disability (YLDs). The relevant formulas were outlined by Murray and are expressed as follows (15, 16):

$$\begin{array}{c} \text{YLLs}[r,K,\beta ]=\frac{KC{\text{e}}^{ra}}{{\left(r+\beta \right)}^{2}}\{{\text{e}}^{-(r+\beta )(L+a)}[-(r+\beta )(L+a)-1]-{\text{e}}^{-(r+\beta )a}[-(r+\beta )a-1]\}+(1-K)/r(1-{\text{e}}^{-rL}). \end{array}$$

The formula for YLDs [*r*, K, β] differs only in the addition of *D* (the disability weight) and is given as follows:

$$\begin{array}{l} \text{YLDs}[r,K,\beta ]=\frac{KC{\text{e}}^{ra}}{{\left(r+\beta \right)}^{2}}\{{\text{e}}^{-(r+\beta )(L+a)}[-(r+\beta )(L+a)-1]-{\text{e}}^{-(r+\beta )a}[-(r+\beta )a-1]\}+(1-K)/r(1-{\text{e}}^{-rL}). \end{array}$$

For the base case recommended and used by Murray and Lopez, discount rate (*r*) = 0.03, age-weighting modulation constant (*K*) = 1, parameter from the age-weighting function (β) = 0.04, and age-weighting correction constant (*C*) = 0.1658 (these all are the standard values given by Global Burden of Disease Study) (14). In case of YLL, age of death (years) is denoted by “*a*” and standard life expectancy at the age of death (years) is denoted by “*L*”. In case of YLD, age of onset (years) is denoted by “*a*” and duration of disability (years) is denoted by “*L*”. Disabilities were assigned severity weights ranging from 0 (representing perfect health) to 1 (representing death) and were adapted from the table given by Murray in 1996 (14, 15, 17).

### Alcohol Consumption and Motorbike Accident

Anecdotal evidences suggested that alcohol consumption while driving the motorbike is one of the major factors to determine the motorbike accident (18). It means if there is a change (such as decrease in percentage change) in alcohol consumption behavior, it will reduce the number of (or percentage change in) motorbike accidents. From the survey data, the ratio of percentage change in alcohol consumption and percentage change in motorbike accident was estimated. The ratio was used to estimate possible effects of reducing motorbike accidents due to the reduction of alcohol consumption.

### Quality Control

Questionnaire was pretested to ensure its suitability and feasibility, and necessary adjustments were made. Field researchers were given 2 days of orientation training where purpose of study, tool being used, and techniques of administering questionnaire were discussed. They were monitored while practicing and immediate feedback was also given to ensure proper data collection. Questionnaire was translated into Nepalese language and retranslated into English in order to assess whether same meaning was retained or not.

### Ethical Consideration

Ethical clearance was obtained from Nepal Health Research Council (NHRC). Informed verbal consent was taken as it was assumed that taking written consent was not feasible from all the participants considering their literacy level. Information sheet was prepared, and it was read to all participants prior to interview. The participants who agreed to participate and can do signatures were asked for it, while for those who cannot sign, a tick mark was put by the researcher and study was proceeded based on a procedure approved by NHRC for obtaining the consent. Participation was absolutely voluntary, and confidentiality as well as anonymity of respondents was maintained.

### Potential Biases

Sometimes in lack of proper filing system of medical records in individual level, exact amount of medical expenses could not be calculated. Memory bias or aggregation of information among motorbike death cases by family members and not remembering many details in case of minor injuries could be other potential biases.

### Limitation of the Study

As the costs calculated in the study were totally based on reporting by victims, there might be underestimation or overestimation of the cost. All the calculations relied on self-reports of respondents. However, wherever feasible and available, bills and records were reviewed. This study did not include travel time cost lost by other travelers and intangible cost (pain, suffering, etc.) associated with accidents.

## Results

From the table, it is evident that trauma occurred between the age of 18 and 53 years with majority being in the age range of 25–40 years, i.e., young adult. Victims were predominantly males (79%), and three-fourths (75%) of respondents were Brahmins/Chhetri. Nearly half of the respondents were engaged in service (49%) followed by student accounting to 27%. Nearly one quarter of victims were found to be drinking alcohol while driving (21%), and road condition was the most commonly reported reasons for accidents (41%) followed by carelessness (21%) (Table 1).

**Table 1:** **Sociodemographic characteristics of respondents (*n* = 100)**.

Variables	Frequency (%)
**Age in years**	
Youth (15–24)	35 (35%)
Young adult (25–40)	46 (46%)
Middle age adult (41–60)	19 (19%)
Range, mean age (SD)	18–53, 30.5 (9.5)
**Gender distribution**	
Male	79 (79%)
Female	21 (21%)
**Ethnicity**	
Brahmin/Chhetri	75 (75%)
Newar	14 (14%)
Thapa/Magar	11 (11%)
**Occupation**	
Service	49 (49%)
Business	19 (19%)
Student	27 (27%)
Others	5 (5%)
**Alcohol consumption while driving**	
Yes	21 (21%)
No	79 (79%)
**Reasons for accident**	
Road condition	41 (41%)
Violation of traffic rules	20 (20%)
Vehicle condition	8 (8%)
Carelessness of other riders/pedestrians	21 (21%)
Overspeed/overtake	10 (10%)

### Patterns of Injuries Associated with Motorbike Accidents in Kathmandu Valley

**Figure 1:**
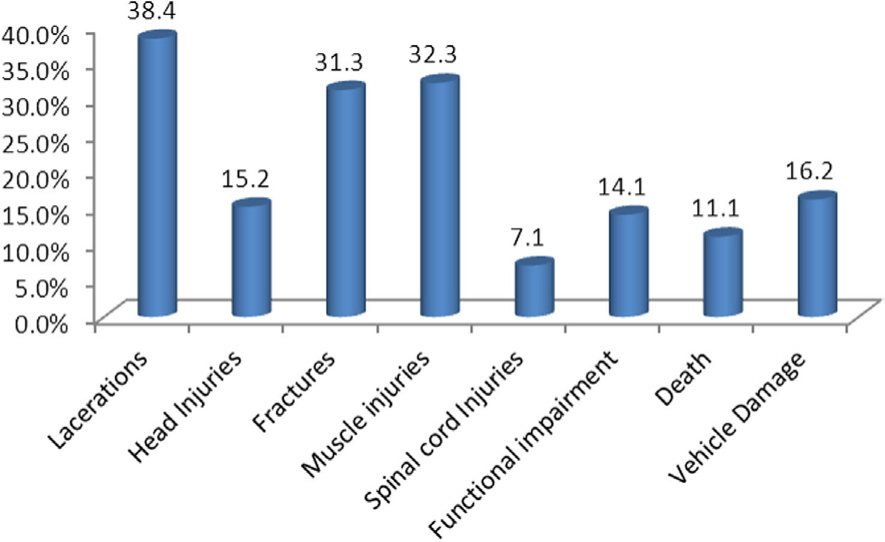
**Patterns of injuries associated with motorbike accidents in Kathmandu Valley**.

Victims had suffered and/or are suffering from multiple injuries due to the accidents. Majority of the victims had lacerations (38.4%), followed by muscle injuries accounting 32.3%. Mortality was found in 11% of cases and 14% of cases have different types of functional impairments such as difficulty in walking and confused mental state (Figure 1).

**Figure 2:**
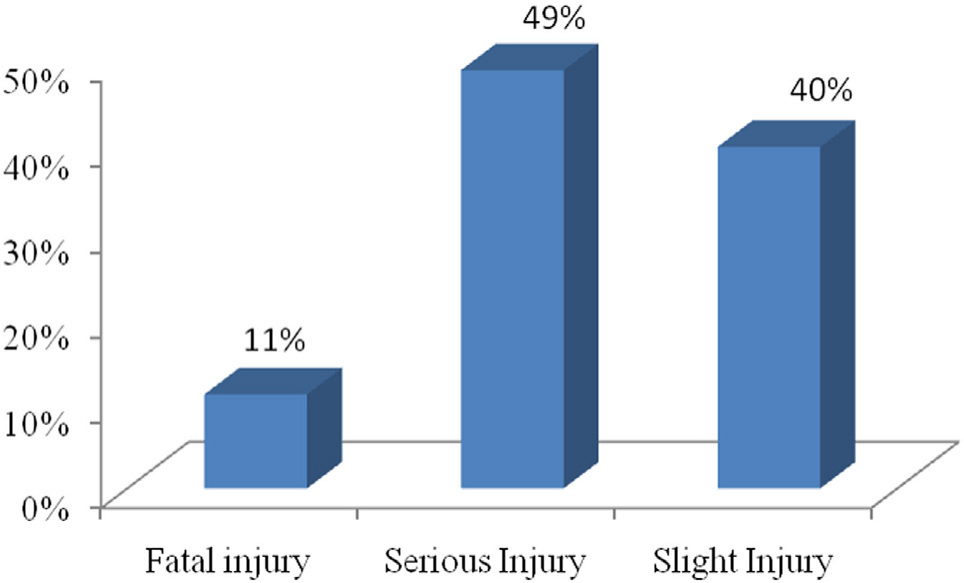
**Types of injuries sustained in motorbike accidents**.

Figure 2 represents the proportion of different types of injuries sustained in motorbike accidents. It is seen that fatalities constituted the lowest proportion, while in majority of cases, there are serious injuries (49%).

### TC Associated with Motorbike Accidents According to Types of Injury

On average, single slight injury cases required Rs. 27,624.13 (US$ 263.79) and for fatal injury Rs. 10,818.18 (US$ 103.31) as direct cost. In case of productivity loss, in a single incidence of injury of serious nature, the victim and his family lost an average of Rs. 31,852.63 (US$ 304.17,) and in case of slight injury, it costs Rs. 4,490.72 (US$ 42.88). Accounting both direct and ICs, on an average, a single accident of serious nature incurs Rs. 121,579.6 (US$ 1,161) and accident of minor injury costs Rs. 32,114.85 (US$ 306.67). About four-fifths of TC is due to direct cost involved in accidents in case of victims who have survived the accident. However, in case of fatal injury, a larger portion of TC is borne by IC and productivity loss due to fatalities was found to be Rs. 101,637.71 (US$ 970.57) per person per annum on an average. Average life lost due to fatal accident was calculated, and it was found to be around 36 years per individual. Proportion of direct cost was minimum (9.62% of TDC) in case of fatal injury compared to other injuries (86.02% in case of slight injury and 73.80% in case of serious injury) (Table 2) (1 US$ = NRs 104.72, Nepal Rastra Bank Exchange Rate, 01/11/015).

**Table 2:** **Total cost (TC) associated with motorbike accidents according to types of injury**.

Types of costs	Types of injuries			Total
	Slight injuries (*n* = 40)	Serious injuries (*n* = 49)	Fatal injuries (*n* = 11)	
Average total direct cost	27,624.13 (86.0%)	89,726.94 (73.8%)	10,818.18 (9.6%)	128,169.3
Direct medical cost	10,151.63	51,963.88	1,136.36	63,251.87
Non-direct medical cost	17,472.5	37,763.06	9,681.82	64,917.38
Total indirect cost (IC)	4,490.72 (14%)	31,852.63 (26.2%)	NA	36,343.35
IC of victims	3,947.80	22,210.11	NA	26,157.91
IC of care takers	542.92	9,642.52	NA	10,185.44
Productivity loss (in case of fatalities)^a^			101,637.71 (90.4%)	
TC	32,114.85	121,579.6	112,455.9	

*^a^Expressed in cost per person per annum*.

*Figures in parenthesis indicate column percentages*.

*Average costs are given in all cases and all costs are expressed in Nepali rupees*.

### DALYs Lost Due to Motorbike Accidents

Disability-adjusted life years is used to show the overall burden of disease and is a health gap measure for disease and injury. One DALY represents the loss of 1 year of equivalent full health. It was found that 364.01 years of life was lost and years lived with disability constituted to be 126.82 years. This gives total DALYs as 490.83 years per person, which shows the gap between current health status and an ideal situation where everyone lives into old age (expectancy life expectancy) free of disease and disability (Table 3).

**Table 3:** **Total disability-adjusted life years (DALYs) lost due to motorbike accidents**.

Years of life lost	Years lived with disability	DALYs
364.01	126.82	490.83

Disability-adjusted life years lost due to motorbike accidents is in increasing trend, which highlights the increasing burden of motorbike accidents and huge economic loss for the country. High DALY loss can be hypothesized as large number of premature deaths and disabilities was due to accident or injury. Large number of DALYs lost due to motorbike accidents supported that it is an economic problem that can have enormous impact on the nation’s economy (Table 4).

**Table 4:** **National estimation of disability-adjusted life years (DALYs) loss due to motorbike accidents**.

Fiscal year	No. of motorcycles involved in accident in Nepal	DALYs lost per year (national estimates) in years
2071–2072 (2014–2015)	5,403	26,529
2070–2071 (2013–2014)	2,144	10,527
2069–2070 (2012–2013)	5,232	25,689
2068–2069 (2011–2012)	4,650	22,832
2067–2068 (2010–2011)	5,544	27,221

*Source: Nepal traffic records, DoHA, GON (2015)*.

**Figure 3:**
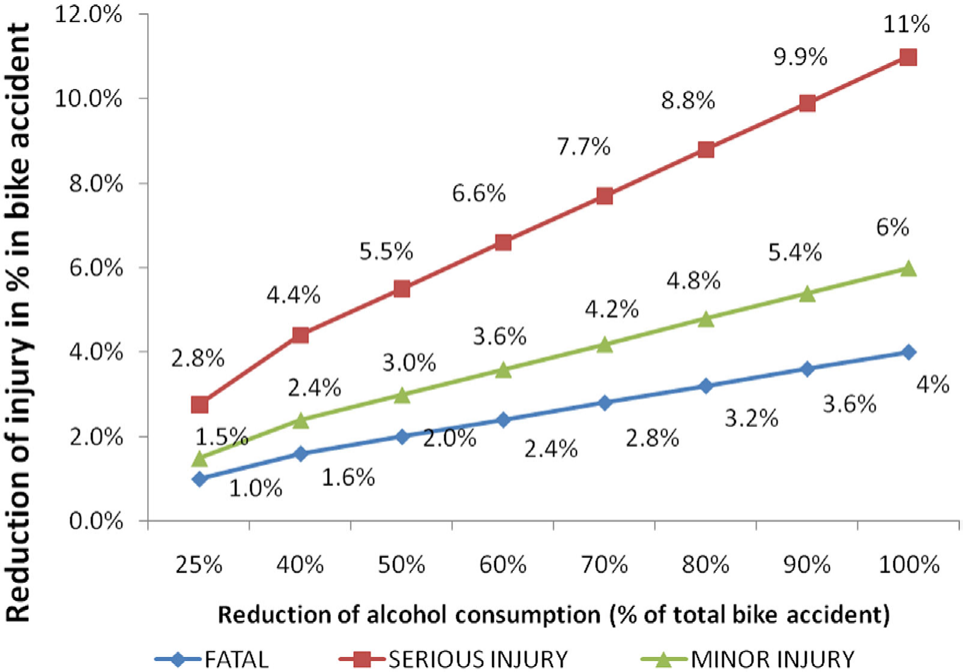
**Reduction of injury due to reduction of alcohol consumption**.

Estimation in accidents reduction was made from the collected data. The result suggested that 21% of the motorbike accidents were due to alcohol consumption. In other words, if there is 100% reduction in alcohol consumption, it will reduce the 21% of motorcycle accidents among which 11% is the proportion of serious injury, 6% of minor injury, and 4% is that of fatal injury. If alcohol consumption is reduced by 25%, there will be 5.3% reduction in motorbike accidents. Similarly, around 15% motorbike accident can be reduced through 70% reduction in alcohol consumption. Large number of fatal and serious injuries can be averted if the alcohol consumption during accident is controlled (Figure 3).

## Discussion

This study examined different types of costs incurred by person and their families involved in motorbike accidents. In consistent to other studies, in this study too, majority of people of productive age group were involved in road traffic crashes. This predominance is evident in studies conducted in other countries of the world as well (8). Males outnumbered females with the proportion of male casualties reaching as high as 80%, and it reflects the reality that if current trend continues, more adult young men in economically productive group will continue to die or get injured as a result of accident, thus reducing productivity. It can have enormous negative economic consequences to the families and nation as a whole. This finding is in accordance with global findings as well as findings from several countries (4, 8, 18). Furthermore, it can be hypothesized that males are more involved in risk-taking behavior such as alcohol consumption, mobile in nature, and engage in outdoor activities more than their female counterparts resulting in heightened risk of accidents. These findings are similar to other studies (9, 19, 20), which showed involvement of alcohol in 20–25% of cases. As a study by Michael Kudebong, a large chunk of accident victims were students suggesting that preventive interventions need to be targeted in young adult males (4). Several causes of accidents identified in this study go in agreement with findings given in paper by Thapa (6, 10), where negligence of the driver stood as the primary cause, followed by high speed and overtaking. It stresses the persistence of negligence and violation of traffic rules by drivers and pedestrians and dire need of strict investigation, law enforcement, awareness program, and punishment in case of any violation.

Mortality from RTA constitute about 25–40% of total deaths in developed countries and 10–30% in developing countries (8, 21). Though fatalities in motorbike accidents are high in this study, the results cannot be directly compared with other findings as it includes fatalities due to all types of accidents.

Similar to our findings, hospital-based studies from Nepal showed fracture as the most common injuries sustained during accidents followed by muscle injuries (7, 22). This study showed large variation in costs as reported in the previous national and international studies. However, it confirms the significant financial burden to victims, family, and community. The conservative cost of road traffic crashes was high, and in case of survival of the victim, direct costs occupy the biggest chunk of TCs accounting to almost 86% which was in agreement with the study done in Barcelona (23). However, ICs in terms of productivity loss outweigh direct costs in the case of disabilities and treatment, highlighting its huge long-term impact on economic welfare of nation. Furthermore, as this study was based on recall, people might have forgotten the costs they have paid in hospitals resulting lower estimation. Another probable explanation for these variations in costs may be difference in health-care delivery systems, insurance pattern, service costs, driving culture, and many other factors across countries.

Economic cost calculated for motorbike accidents are just the tip of iceberg of total individual killed, crashed or disabled by motorbike accidents, and there is higher cost associated with the expenses of prolonged medical care, loss of family breadwinner, or the added burden of caring for the disabled. Consistent to our study, a study done by Miller et al. showed that total medical costs account for 6% of TCs of motorcycle injuries, of which 29% is due to work loss (24).

This study is limited to small sample size due to communication and financial constraints and lack of proper recording mechanism. Despite its small sample size, it is the first study of its kind in our setting and provides useful information regarding economic burden, which can be used as a reference for conducting further researches and program designing. Even though productivity loss accounts for huge burden to victims, families, and society, it is unrecognizable yet. This study attempts to show the economic burden by including these indirect losses also and hence highlight the appropriate recognition and awareness of a whole society to traffic safety aspect. The cost estimation in this study is of minimum value as some of the cost elements have not been included due to the lack of information, and it does not include intangible cost such as pain grief, suffering, household work loss, and travel time loss due to accidents. Use of non-random sampling might have introduced selection bias, and attempt was made to select respondents as randomly as possible.

It is recommended to conduct national costing of road accidents immediately to address emerging problem of accidents, its associated consequences, and worsening situation of road safety. There is a strong need of adopting effective policy interventions for reducing the number of motorbike accidents for the improvement of overall community’s health status through reduction of life years lost and years living with disabilities. Strict enforcement of policy-related motorcycle-safety measures such as regular helmet use and driving license will contribute a lot to reduce motorbike accidents. Furthermore, it will reduce imposed costs on the health system and country’s economies as study showed that accident impose a high financial burden, directly and/or indirectly. Findings suggest that burden of RTC can be catastrophic for families because of high direct costs, significant absent days from work or schools for patients as well as caregivers, and impact on health-related quality of life. The importance of the problem warrants further interventions to reduce motorbike crashes.

## Conclusion

This study concluded the persistence of RTC as significant public health problem from economic lens. Costs of motorbike crashes are very high both in terms of epidemiological and economic aspects. All components of cost were significantly high with direct costs constituting largest proportion in case of serious injuries and IC in case of fatal injuries. The increasing burden of motorbike accidents and large amount of economic cost associated with it calls for considering appropriate research and effective interventions. Relatively high loss of healthy life from accidents needs to be addressed by public health systems in Kathmandu. There should be strict enforcement of law regarding regular helmet use, issuing driver’s license, and regular mass education on high economic burden of motorbike crashes. Record mechanism in traffic police and hospital sections need to be strengthened with inclusion of all the details of accident.

## Author Contributions

DS and SA were involved in conception and design of the study. DS was involved in tool preparation. DS, BB, and SA edited and finalized the tools. DS and BB were involved in data acquisition and in analysis and interpretation. All the authors participated in report writing and providing intellectual input. DS and SA prepared the draft that was critically reviewed and approved by all the authors. The manuscript has been read and approved by all the authors, and the requirement for authorship was fulfilled by all authors.

## Conflict of Interest Statement

The authors declare non-existence of financial relationships with any entities that could influence the submitted work. There is no competing interest.

## Abbreviations

ADB, Asian Development Bank; DALYs, disability-adjusted life years; DMC, direct medical cost; DNC, direct non-medical cost; FY, fiscal year; GDP, gross domestic product; GNP, gross national product; IC, indirect cost; INEHD, Institute for Nepal Environment and Health System Development; NA, not available/not applicable; PDO, property damage only; RTA, road traffic accidents; RTI, road traffic injuries; TC, total cost; TRL, Transport Research Laboratory; WHO, World Health Organization; YLDs, years lived with disability; YLLs, years of life lost.
